# Reflections and Practical Insights on Communication and Care for Patients and Families in Japanese Intensive Care Units During COVID-19: A Semi-structured Interview Study of Healthcare Providers

**DOI:** 10.1007/s41649-025-00402-z

**Published:** 2026-04-25

**Authors:** Yusuke Seino, Atsushi Kogetsu, Kazuto Kato

**Affiliations:** 1 Department of Biomedical Ethics and Public Policy, Graduate School of Medicine, Osaka University, Osaka, Japan; 2 Department of Healthcare Ethics, Kyoto University School of Public Health, Kyoto University, Kyoto, Japan

**Keywords:** Communication, COVID-19, Family care, ICU, Interdisciplinary collaboration

## Abstract

This study explored challenges experienced by healthcare providers (HCPs) in intensive care units (ICUs) during the COVID-19 pandemic, focusing specifically on communication and care interactions, and examined how these experiences impacted ICU practices and provided opportunities for improvements. Semi-structured interviews were conducted with 16 ICU physicians and nurses from October 2022 to March 2023. Interview data were analyzed through thematic analysis. Nine themes emerged, categorized into three key areas. (1) “Challenges and Adaptations Experienced by HCPs” described how stringent infection-control measures created emotional and practical difficulties, prompting adaptive strategies to maintain effective care. The pandemic reinforced the critical role of interdisciplinary cooperation; in response, an interdisciplinary conference was organized, and HCPs intentionally sought closer collaboration than was usual to ensure coordinated decision-making and the continuity of care. (2) “Interactions Among Patients, Families, and HCPs” emphasized that visitation restrictions impaired family involvement, although proactive measures by nurses, such as the introduction of remote visits, significantly facilitated family engagement and emotional support. (3) “Lessons Learned from the Pandemic” highlighted improved healthcare practices and reinforced the value of interdisciplinary collaboration and family-centered approaches in ICU care. The findings underscore the necessity of robust communication practices and interdisciplinary teamwork within ICUs. These pandemic-related experiences offer valuable insights for improving communication, supporting flexible visitation policies, and promoting patient- and family-centered approaches. Collectively, these lessons offer essential guidance for developing more resilient and compassionate ICU care strategies, applicable in future crises and in everyday practice.

**Supplementary Information:**

The online version contains supplementary material available at 10.1007/s41649-025-00402-z.

## Background

Effective communication among healthcare providers (HCPs), patients, and their families is crucial for delivering quality medical care to critically ill individuals in intensive care units (ICUs). However, achieving effective communication remains challenging due to factors such as multiple stakeholder involvement, rapid fluctuations in patient conditions, and ethical complexities, particularly when addressing life-threatening situations (Grant 2015; Vincent 1997).

Recently, the role of patients’ families in critical care has received increasing recognition. Families provide emotional support, assist in shared decision-making, and advocate for patient needs (Chen et al. 2021). Nevertheless, family members themselves are at risk for psychological complications, including anxiety, depression, and post-traumatic stress disorder, collectively referred to as post-ICU syndrome family (PICS-F) (Davidson et al. 2012). Evidence indicates that supporting families can enhance health outcomes for both patients and their family members (Davidson et al. 2012; Needham et al. 2012).

Family-centered care (FCC), which emphasizes partnerships among HCPs, patients, and their families during the planning, delivery, and evaluation of healthcare, has gained prominence (Davidson et al. 2017). In 2017, the Society of Critical Care Medicine published guidelines on FCC to enhance family support practices, marking a significant step toward improved global practices in ICU care (Davidson et al. 2017).

The COVID-19 pandemic, first reported in December 2019, has significantly impacted global healthcare, particularly in ICUs, where surges in patient admissions and intensified infection-control protocols have placed considerable strain on HCPs (Kader et al. 2021; Wahlster et al. 2021; Kentish-Barnes et al. 2021). Strict visitation restrictions impaired communication, causing emotional distress among patients, families, and HCPs (Digby et al. 2023a, b); Vranas et al. 2022; Krewulak et al. 2022; Onwuteaka-Philipsen et al. 2021; Seino et al. 2022). The exclusion of family members disrupted the implementation of FCC, severely affecting patient and family support (Rose et al. 2021; Fernández-Martínez et al. 2022; Hart et al. 2020). Similar challenges emerged in Japan, where increased infection-control measures restricted communication and caused psychological distress among ICU providers, complicating patient-centered decision-making and compromising patient and family care (Seino et al. 2022; Nishimura et al. 2023; Kuriyama et al. 2023; Matsuda et al. 2021). However, limited research has examined how ICU HCPs coped with communication and caregiving challenges during the COVID-19 pandemic or how their experiences shaped approaches toward interdisciplinary collaboration and family-centered care in the post-pandemic context (Kentish-Barnes et al. 2021; Fernández-Martínez et al. 2022).

This study explored the communication and caregiving challenges experienced by ICU HCPs during the COVID-19 pandemic. Additionally, it examined strategies utilized to address these difficulties and the insights obtained, aiming to enhance future communication and caregiving practices both during crises and in routine ICU care. We aimed to contribute toward the development of a deeper understanding of how pandemic-related experiences transformed everyday communication, teamwork, and family involvement in intensive care. Furthermore, the study findings can provide practical implications for developing more resilient, adaptive, and family-centered ICU practices in the post-pandemic era.

## Methods

This qualitative descriptive study explored the experiences of HCPs working in ICUs in Japan during the COVID-19 pandemic. Data were collected via semi-structured interviews and analyzed using thematic analysis.

### Participants

Participants included ICU physicians and nurses providing COVID-19 care between March 2020 and March 2022. In this study, we selected physicians and nurses as interview participants because they were the primary HCPs who were directly responsible for communication and care in ICUs during the COVID-19 pandemic. These two groups were at the frontlines of coordinating patient care and interacting with patients and families, making their perspectives highly relevant to the study’s aims. Using purposive sampling, healthcare facilities were recruited from the Kansai region, an area significantly affected by the pandemic and central to severe COVID-19 care. Participants were identified through snowball sampling via facility leaders. Inclusion criteria were ICU providers with at least 2 years of ICU experience; no age restrictions were applied. Informed consent was obtained from each participant before the interviews.

### Research Method

Semi-structured interviews, approximately 60 min each, were conducted between October 2022 and March 2023 using an interview guide (Table 1). Participants selected their preferred interview format (online or in-person). Although 16 interviews were initially planned, data collection and analysis were concluded when thematic saturation was achieved, that is, when no new themes or subthemes emerged from the data, as confirmed through discussion among the research team. The interview guide was reviewed by co-authors (KA and KK) for clarity and relevance to the research questions, followed by a pilot interview. Participants were informed that they could skip uncomfortable topics or withdraw at any point during the study. Ethical approval was obtained from the Ethics Review Committee of Osaka University (Ethics Review No. 22138).

**Table 1:** Interview guide

(1) Participant Background Sex, age, and years of ICU experience, experience in other ICUs, current ICU position, percentage of workload involving patients with COVID-19, number of patients with COVID-19 treated
(2) How did you feel when you began caring for critically ill patients with COVID-19 in your ICU?
(3) What was the most challenging aspect of caring for patients with COVID-19 in your ICU?
(4) Were visitation restrictions implemented in your ICU? a: Were there differences in restrictions over time? b: Were visits allowed based on end-of-life status or other patient conditions? c: What communication methods were available between patients and families during visitation restrictions? d: How did healthcare providers communicate with families during visitation restrictions?
(5) Did you experience difficulties or stress in communication with patients with COVID-19 during the pandemic? a: In what situations did these difficulties occur?b: What specific challenges did you face?c: How did you attempt to address these challenges? d: Was there collaboration or consultation among healthcare providers within or across specialties, or were there conflicts or disagreements? e: Do you have thoughts on the division of roles and collaboration among healthcare providers regarding patient communication? Did your perspective change before and after treating patients with COVID-19? f: In hindsight, how do you think you should have responded? Are there differences between the early stages of the pandemic and now?
(6) Did you experience difficulties or stress in communication with the families of patients with COVID-19 during visitation restrictions? a: In what situations did these difficulties occur? b: What specific challenges did you face? c: How did you attempt to address these challenges? d: Was there collaboration or consultation among healthcare providers within or across specialties, or were there conflicts or disagreements? e: Do you have thoughts on the division of roles and collaboration among healthcare providers regarding communication with families? Did your perspective change before and after treating patients with COVID-19? f: In hindsight, how do you think you should have responded? Are there differences between the early stages of the pandemic and now?

The interview transcripts were analyzed using Braun and Clarke’s six-phase method of reflexive thematic analysis (Braun and Clarke 2006), which included familiarization with data, generation of initial codes, search for themes, review of potential themes, definition and naming of themes, and production of the report. The themes were derived inductively with a semantic focus, addressing the research questions and goals of the study. Coding was performed by the author (SY); the coding process, subthemes, themes, and categories were discussed and refined collaboratively with the research team (SY, KA, and KK) through consensus. To enhance transparency, representative examples of coded quotations illustrating the analytic process are included in Supplementary Table 1. This approach ensured that the themes reflected words and concepts relevant to the research questions and enhanced the rigor of the analysis.

### Reflexivity

All interviews were conducted by the first author (SY), an experienced intensivist. Although SY did not work in the same institutions as the participants, sharing the same professional background could have influenced interactions during interviews and the interpretation of data. To address this potential bias, SY remained aware of her own preconceptions and continuously reflected on how her professional perspective might shape data collection and interpretation. The analysis was conducted in close consultation with co-authors (KA and KK), who contributed diverse clinical and research perspectives. The co-authors reviewed coding, themes, and interpretations, helping to identify blind spots and minimize bias. This process of analytical triangulation strengthened the trustworthiness of the findings.

## Results

**Fig. 1:**
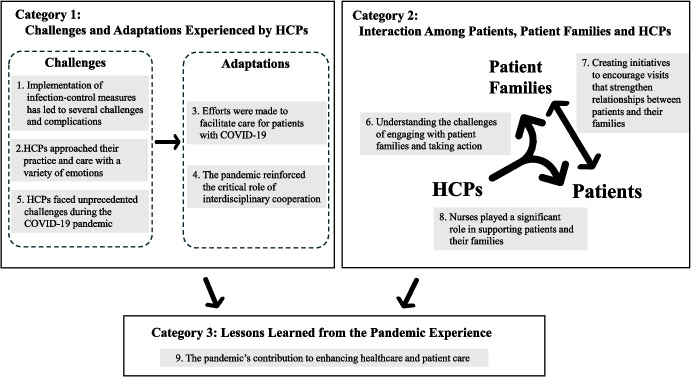
Relationships among the main themes

Sixteen participants completed the interviews, all preferring remote, web-based formats. The participants included eight physicians and eight nurses, with seven (43.7%) identifying as male (Table 2). Participants had an average ICU experience of 10.6 years, and four (25%) held leadership positions. All participants were recruited from four different healthcare facilities. Collectively, participants reported managing an average of 187 patients with COVID-19 during the study period. Based on the interview results and using thematic analysis, three categories, nine themes, and forty subthemes were developed. Figure 1 presents a diagram illustrating interrelationships among the main themes identified in the analysis. All participants contributed to multiple themes, and no substantial imbalance was observed in the distribution of responses.

**Table 2:** Demographic profile of participants

No.	Profession	Sex	Age	Years of ICU experience	Experience in other ICUs	Position in the ICU	Number of patients with COVID-19 treated
D-a-1	Doctor	Male	30s	7	No	Staff	300
D-a-2	Doctor	Male	40s	13	Yes	Chief	200
N-a-3	Nurse	Male	20s	7	No	Staff	100
N-a-4	Nurse	Female	30s	5	No	Staff	40
N-b-1	Doctor	Male	40s	14	No	Chief	500
D-b-2	Doctor	Female	30s	12	No	Staff	200
N-b-3	Nurse	Female	50s	13	Yes	Chief	100
N-b-4	Nurse	Male	30s	12	Yes	Staff	100
D-c-1	Doctor	Female	30s	9	No	Staff	100
D-c-2	Doctor	Male	40s	12	Yes	Staff	150
N-c-3	Nurse	Female	20s	6	No	Staff	300
N-c-4	Nurse	Female	40s	9	No	Staff	50
D-d-1	Doctor	Female	50s	21	Yes	Staff	250
D-d-2	Doctor	Male	40s	7	No	Chief	250
N-d-3	Nurse	Female	30s	11	No	Staff	260
N-d-4	Nurse	Female	30s	12	Yes	Staff	100

### Category 1: Challenges and Adaptations Experienced by HCPs

#### Theme 1. The Implementation of Infection-Control Measures has led to Several Challenges and Complications

HCPs faced significant challenges when caring for patients in COVID-19-designated areas, particularly related to the mandatory use of personal protective equipment (PPE). PPE usage contributed to patient isolation, reduced direct patient-care time, and delayed responses, subsequently increasing patient delirium and staff-related incidents. A participant noted, “*Instead of us struggling, I felt the patients were suffering, as PPE makes it hard to read expressions*” (D-a-2), highlighting concerns about increased patient anxiety. Adaptations to address these issues included utilizing walkie-talkies for improved communication and installing cameras for enhanced patient monitoring. Moreover, PPE complicated auditory communication, prompting HCPs to speak more loudly and clearly.

Additionally, restrictions preventing direct interaction between HCPs, patients, and families impaired the collaborative discussions crucial for care planning. A participant explained, “*Normally, HCPs, patients, and their families can gather at the bedside, but the pandemic interrupted this, making communication uniquely challenging*” (N-b-4). Another participant stated, “*Even if we communicated *via* smartphones, it was difficult to foster a sense of togetherness during discussions. The indirect sharing of information felt frustratingly detached*” (D-b-1).

#### Theme 2. HCPs Approached their Practice and Care with a Variety of Emotions

Participants expressed varied emotional responses toward providing COVID-19 care. Initially, they reported little hesitation, viewing their involvement as fulfilling their professional and social responsibilities. Despite their motivation to provide the same quality of care as in non-pandemic times, HCPs faced significant psychological stress and ethical tension, largely due to restrictive infection-control measures enforced by hospital policies and interprofessional disagreements. Additionally, visitation restrictions prevented family members from supporting patients directly, prompting HCPs to adopt supportive roles traditionally fulfilled by families. One participant explained, “*With COVID-19, the inability of families to visit meant that there were tasks they could not perform. I aimed to engage with patients as if I was taking that care instead of families, and I believe that sentiment has grown stronger*” (D-a-4). Another reflected, “*I realized that we, as HCPs, must interact with patients sincerely. While I am not exactly replacing family*…” (D-d-1).

HCPs also experienced conflicts and dissatisfaction arising from disagreements among medical professionals, particularly regarding critical treatment decisions. Differences in perspectives between primary physicians and intensivists led to frustration, notably when some physicians hesitated to enter patient rooms due to fears of infection. Participants additionally reported anxiety, stress, and feelings of powerlessness associated with the unprecedented challenges and inadequate preparation for pandemic-specific situations.

#### Theme 3. Efforts were made to Facilitate Care for Patients with COVID-19

Healthcare facilities adopted multiple strategies to address challenges related to COVID-19 care. Establishing workload-sharing systems among hospitals and developing standardized treatment guidelines substantially alleviated frontline stress. Centralized care and clarified roles between neighboring hospitals improved efficiency, although balancing routine hospital functions with pandemic care remained challenging.

ICU treatment processes were simplified, enabling fewer staff to manage more patients effectively. Several facilities created standardized manuals to facilitate communication with patients’ families. One participant noted,“*Recognizing that individual HCPs could not sustain prolonged patient care, we standardized manuals for intubation, extubation, and management before initiating COVID-19 admissions.*” (D-b-1). Another stated, “*As various HCPs became involved in COVID-19 care, detailed treatment manuals gradually emerged*” (D-c-1).

While standardized manuals improved interdepartmental collaboration, communicating protocols to newly assigned support physicians occasionally caused additional stress. Rotating shifts among staff also disrupted consistent relationships with patients’ families. During resource shortages, hospital leadership conferences guided resource allocation decisions.

#### Theme 4. The Pandemic Reinforced the Critical Role of Interdisciplinary Cooperation

All participants emphasized the importance of interdisciplinary collaboration. Intensivists managed patient care, while attending physicians oversaw key treatment decisions and communicated with families, reflecting effective collaboration across specialties. Specialists regularly exchanged patient information to minimize conflicts and bridge communication gaps. Interdisciplinary conferences were organized to facilitate more effective communication with patients’ families. Additionally, detailed instructions were provided proactively, reducing nurses’ need for repeated clarification and streamlining patient care procedures.

Participants noted that the pandemic fostered a stronger collaborative spirit: “*It felt like we were starting COVID treatment from scratch together, and I found it easier to consult with different professions during this time*” (N-d-3). One participant stressed communication’s significance: “*If we don’t prioritize this, it could lead to serious issues*” (D-b-1). Before contacting families, HCPs exchanged critical information: “*I would always check with the physician about the tone and atmosphere of their explanation since the nurse was not present*” (N-b-4).

Many participants described positive experiences from interdisciplinary collaboration, highlighting conferences as essential for aligning team efforts. Family support teams also improved interactions with families: “*Since the family support team started attending morning briefings, the exchange of information with patients’ families has significantly increased compared to when we handled it alone*” (D-b-1). Additionally, participants recognized that diverse professional perspectives enriched patient care discussions: “*The conference provided great insights; it was crucial to gather diverse opinions on a single patient*” (N-b-3).

However, participants also reported instances of inadequate collaboration. In some cases, physicians explained patient conditions to families without consulting nurses, and attending physicians rarely visited wards, restricting opportunities for communication. Limited physician engagement in patient care resulted in discrepancies between nurses’ and physicians’ perceptions of patient conditions.

#### Theme 5. HCPs Faced Unprecedented Challenges during the COVID-19 Pandemic

Participants described encountering unprecedented challenges during the COVID-19 pandemic. As understanding of COVID-19 evolved, clinical management improved, yet emotional and psychological needs intensified. Many patients expressed guilt toward their families for contracting the virus, underscoring the necessity for psychological support that was provided by clinical psychologists and psychiatric nurses.

HCPs reported consistent patient communication despite barriers posed by PPE. Increased nursing staff facilitated effective care delivery. Physicians also highlighted the ethical complexity of treatment decisions, noting the necessity for earlier decision-making and psychological support from clinical psychologists and psychiatric nurses. One participant stated, “*Many patients felt guilty toward their families, indicating a critical need for psychological support*.”

However, challenges persisted, including restricted communication due to PPE and psychological distress among patients. Additionally, decisions regarding critical care were sometimes made prematurely. Participants reported the importance of psychiatric support to address these issues. Despite these constraints, communication remained consistent, largely due to increased nursing staff, facilitating effective patient care.

### Category 2: Interactions Among Patients, Families, and HCPs

#### Theme 6. Understanding the Challenges of Engaging with Patient Families and Taking Action

Visitation restrictions made it challenging for families to clearly understand patients’ conditions and ongoing treatments. Explanations provided remotely by HCPs were often insufficient, leaving families struggling to fully grasp patients’ situations. One participant explained, *“Due to visitation restrictions, families unable to see their loved ones have found it difficult to envision the ongoing treatments. In addition, explanations from HCPs *via* telephone or web communication have often failed to convey the necessary information effectively.” Another participant noted, “The more serious the patient, the harder it is for the family to understand. It was always a struggle. Hearing about the treatment over the telephone made it difficult for families to fully understand the situation.” (D-c-1).* A HCP observed that, “*regardless of the thoroughness of verbal explanations, families often experienced shock upon seeing the patient*’*s condition firsthand*” (N-d-4). To address these challenges, visual communication methods, including remote video meetings, were implemented to help families better understand the patients’ status, allowing them to see not only the patients but also their surrounding environment, such as respiratory devices. However, this method did not entirely replicate the experience of direct visits. “*Viewing a patient on a ventilator through an online platform can be beneficial, but the family may not completely understand the seriousness of the situation*” (D-b-2). Additionally, “*Online platform communication introduced challenges, including time lags and difficulty perceiving emotional nuances*” (N-b-4). Efforts continue to refine these communication approaches to better support emotional engagement between patients, families, and HCPs.

Participants also expressed concern that automated camera adjustments could inaccurately portray patient conditions, complicating families’ understanding. Telephone communication further hindered accurate interpretation, as it limited HCPs’ ability to assess families’ emotional reactions. A participant highlighted, “*Communication via telephone complicated the ability to read family members’ expressions, making it difficult to understand their emotional state accurately*” (D-b-1). Differences between physicians’ and nurses’ perceptions of families’ emotional states also presented challenges.

Despite these issues, telephone communication was necessary when direct interactions were restricted. Participants noted significant limitations, particularly in scenarios involving multiple family members. One explained, “*Telephone communication was especially challenging in group discussions requiring multiple family members’ involvement*” (D-b-1). To mitigate these barriers, healthcare teams prioritized direct interactions whenever possible, emphasizing their value in sensitive family discussions.

To better address these communication challenges, many facilities introduced family support teams dedicated to providing emotional support and updates. This intervention facilitated clearer, more consistent communication with families unable to visit in person, improving mutual understanding of the patient’s condition and treatment plan. Furthermore, “*A physician noted the limitations of telephone communication, particularly in group settings where multiple family members needed to be involved*” (D-b-1). Consequently, it was reported that key discussions with families were conducted through direct interaction whenever possible.

#### Theme 7. Creating Initiatives to Encourage Visits that Strengthen Relationships between Patients and their Families

Owing to infection-control measures, hospital visitation was highly restricted, creating emotional distress among patients and families. One participant reflected, “*I wanted to find ways for families to visit, but continuously* m*a*k*ing exceptions felt inappropriate for the organization*” (D-a-2). Differences also emerged among HCPs; while physicians tended to favor restrictions due to infection risks, nurses were more inclined to advocate for family presence. Discrepancies in visitation policies between hospitals further complicated the issue.

Remote visits were implemented to maintain patient-family connections. Initially, privacy concerns and the need for clear guidelines created barriers. Additionally, some families avoided in-person visits due to fear of infection. Despite these obstacles, families expressed a strong desire to visit patients during critical situations, highlighting the emotional importance of direct interaction. As one participant noted, “*When significant circumstances arise, the disconnect in time and space is unsettling. While not everyone needs physical contact, for some, seeing and touching can make a difference*” (D-b-1).

As direct visits resumed, feedback from families indicated relief from being able to see the patients directly. Despite the challenges posed by the pandemic, in-person visits, whether through windows or directly, were preferable to online meetings. Efforts have been made to facilitate these encounters, such as allowing families into negative-pressure rooms or, in end-of-life situations, having family members donate PPE for direct visits with consent. Retrospectively, there is a sense of regret about not swiftly establishing visitation protocols. One participant remarked, “*If we could have devised a system more quickly or found innovative methods for visits, we could have created more opportunities for patients and families to connect*” (N-b-4).

#### Theme 8. Nurses played a Significant Role in Supporting Patients and their Families

Nurses played a central role in supporting families throughout the COVID-19 pandemic. Before the pandemic, nurses participated in family briefings alongside physicians, helping families understand patients’ conditions and encouraging emotional expression. Although direct family visits were suspended during the pandemic, nurses continued to engage families separately from physicians, allowing families to voice concerns they might hesitate to share directly with physicians. Nurses validated these anxieties and served as critical intermediaries, linking families with multidisciplinary support teams.

Participants emphasized nurses’ critical role in connecting HCPs with families. One nurse explained, “*Establishing communication with families is essential. It’s our responsibility to interpret their emotional* states and perceptions, conveying *these to physicians and specialists*. *When specialists are available,* we *connect families to them*” (N-b-4). Another participant highlighted nurses’ growing importance in *team-based care*, noting, “Facilitating *these connections* has become a key nursing *responsibility*” (N-b-4).

### Category 3: Lessons Learned from the Pandemic Experience

#### Theme 9. The Pandemic’s Contribution to Enhancing Healthcare and Patient Care

The pandemic experience significantly raised awareness of interdisciplinary collaboration. Opportunities for interdisciplinary discussions regarding patient-centered goals increased, shifting focus from solely medical management toward care aligned with patient values. Enhanced communication systems facilitated these interactions, notably between physical therapists and nurses. As one participant remarked, “*We increasingly discuss what we can do to support patients in reaching their goals*” (N-a-4). Another emphasized the *educational impact,* stating, “The pandemic *compelled HCPs to consider medical care* necessary *to* help *patients live the lives they desire*” (D-b-1). Structured ethical consultations clarified previously *vague concepts* related to *prognosis and* patient *values*.

*The* onset *of COVID-19 coincided with the hospital’s efforts to* integrate *ethical practices into* routine *operations.* Following *years of establishing an ethics consultation team and training staff* in *ethical* decision-making*, the hospital was well-prepared* when *the pandemic* began (b-1).

Additionally, attitudes toward patients and families evolved, promoting a more patient-centered approach. Participants emphasized that the enhanced *focus on* individual *patient* needs *during COVID-19* positively influenced routine *emergency care beyond* the pandemic. One nurse noted, “It may have been a valuable opportunity to reevaluate routine nursing practices” (N-b-4).

New insights emerged regarding routine medical care. Face-to-face bedside meetings between patients and families demonstrated the importance of physical presence for effectively understanding patient conditions. Participants recognized that traditional *ICU settings often* lacked opportunities for meaningful family interactions, *prompting* a reassessment of visitation policies. As one participant reflected, “COVID-19 prompted *deeper reflections on* how and *when families* could *best visit loved ones*” (N-d-4). “*Looking back, it may have been a valuable opportunity to reevaluate routine nursing practices*” (N-b-4).

## Discussion

This study focused on the impact of the COVID-19 pandemic on communication and medical care between HCPs and patients and their families in Japanese ICUs through interviews with HCPs.

### Unprecedented Challenges in ICU Care During COVID-19

The COVID-19 pandemic presented unprecedented challenges for ICU HCPs. Consistent with findings from other countries (Digby et al. 2023a; Vranas et al. 2022), strict infection-control measures in Japanese ICUs reduced opportunities for meaningful interactions between HCPs, patients, and families. Isolation protocols and PPE significantly limited patient contact and communication time. Despite these constraints, HCPs remained committed to supporting patients as best they could.

The absence of patients’ families in the ICU had the most significant impact. This absence hindered communication and complicated decision-making for terminally ill patients (Hanna et al. 2021). HCPs sought to bridge communication gaps by acting as intermediaries between patients and their families, underscoring the continued importance of patient- and family-centered care. Before the pandemic, visitation policies in Japanese ICUs were relatively restrictive, and family facilities such as waiting rooms were often limited. Therefore, the complete visitation ban during COVID-19 may have had a disproportionately greater impact on family isolation and psychological distress compared with the corresponding impact in settings where family presence had been more routinely supported (Shirasaki et al. 2025).

Participants also reported feeling considerable psychological distress and helplessness as patients’ conditions deteriorated. Despite personal fears of infection, they continued to fulfill their duties out of a sense of professional responsibility. Moreover, with increased clarity about COVID-19 disease progression, HCPs could better align treatment decisions with patient values, although variations in care approaches emerged among physicians.

Patients often experienced guilt toward family members after contracting COVID-19, emphasizing the need for enhanced psychological support. These experiences underscore the complex and unprecedented nature of providing ICU care during the pandemic.

### Strengthening Interdisciplinary Collaboration and the Role of Nurses

While highlighting unprecedented challenges, the pandemic also exposed underlying issues in ICU care. In Japan, concepts such as PICS-F and FCC were included in treatment guidelines even before COVID-19 (Egi et al. 2021), gradually increasing recognition of family support needs. Pandemic-induced communication restrictions heightened HCPs’ awareness of previously routine interactions, prompting them to reassess patient- and family-support practices.

Before the pandemic, Japan’s healthcare system had been shaped by a strongly physician-centered culture, where both HCPs and patients were accustomed to physicians taking primary responsibility for all aspects of care. This hierarchical structure often limited interdisciplinary collaboration and flexibility (Igarashi et al. 2022). However, during the pandemic, this traditional model was difficult to sustain under staff shortages and infection-control restrictions, forcing many ICUs to transition toward more team-based and collaborative care.

To address limited staffing and the challenges posed by infection-control measures, ICUs adopted systemic, interdisciplinary interventions. These changes shifted support for patients and families away from reliance on individual HCP efforts toward embedded team-based care. In Japan, where ethical consultation was already being adopted more widely (Nagao and Takimoto 2024), the ethical stress experienced during the COVID-19 pandemic heightened HCPs’ sensitivity to ethical decision-making. As a result, the role of ethics consultation expanded, thereby strengthening interdisciplinary collaboration.

A review of FCC practices during the pandemic (Fernández-Martínez et al. 2022) also highlighted the usefulness of interdisciplinary interventions. The creation of manuals and rules for on-site implementation and the deliberate effort to hold more frequent and detailed conferences fostered collaboration and cooperation. These experiences of realizing the effectiveness of interdisciplinary collaboration during the pandemic are considered to have contributed to the advancement of interdisciplinary teamwork in Japanese ICUs during normal times as well. Participants particularly emphasized nurses’ essential roles in managing family interactions. Although both physicians and nurses communicate with families, nurses do so with greater empathy and focus on family dynamics, distinguishing their contributions clearly among HCPs. Beyond traditional nursing responsibilities (Sekse et al. 2018), the pandemic highlighted nurses’ role as vital connectors between patients, families, and the healthcare system—a role that will remain central in future ICU care.

### Importance of Direct Patient-Family Visits

Strict visitation restrictions were enforced in healthcare facilities worldwide (Krewulak et al. 2022; Tabah et al. 2022; Azad et al. 2021), and it is known that many hospitals did not permit visits even for patients in the terminal stage (Rose et al. 2021). Although many institutions adopted alternative methods such as phone calls and remote visits (Rose et al. 2021; Kennedy et al. 2021), the facilities of the participants in this study also implemented these measures. Moreover, even though telephone and remote communication were employed to maintain connections between patients and families, participants reported significant limitations with these methods. Families frequently struggled to fully comprehend patient conditions, and HCPs faced difficulties accurately assessing families’ understanding and emotional states.

Participants highlighted that phone and remote communication inadequately replaced face-to-face interactions, which is a view supported by existing literature (Digby et al. 2023a; Rose et al. 2021; Fernández-Martínez et al. 2022; Kennedy et al. 2021). Direct visits were described as crucial, allowing families to better grasp the seriousness of the patient’s condition through firsthand experience. During the pandemic, efforts to facilitate remote connections did not fully compensate for the absence of physical presence, as families could not sufficiently perceive the patient’s condition or hospital atmosphere remotely (Digby et al. 2023a; Nishimura et al. 2023; van Veenendaal et al. 2021).

Participants emphasized the importance of face-to-face interactions, arguing that direct visitation significantly influences the emotional well-being of patients and families. Although infection-control measures remain essential, future pandemic responses should prioritize strategies that support family presence from the beginning. Additionally, as restrictive visitation policies remain common in routine healthcare settings (Nassar Junior et al. 2018), experiences from COVID-19 may encourage a shift toward more flexible visitation practices moving forward.

### Impact of Remote Visitation on Family Description and ICU Practices

The implementation of visitation restrictions during the COVID-19 pandemic introduced “remote visits” as a novel method for maintaining patient-family connections. This technological innovation has allowed patients to communicate effectively with family members who live far away or have mobility limitations, circumstances that make in-person visitation difficult.

The description of patient–family relationships should ideally reflect the quality and closeness of their connections with the patient. However, historically, family members who could physically visit the ICU were presumed to be the primary points of contact. The FCC guidelines emphasize open visitation and encourage family participation in ICU team rounds, highlighting that physical presence is a fundamental element in delivering patient and family-centered care (Davidson et al. 2012). Yet, these guidelines do not specifically address situations involving family members who cannot physically visit the ICU. The introduction of remote visits has significantly expanded HCPs’ perceptions of “family,” prompting them to reconsider their understanding beyond physical presence alone. This shift indicates that the meaning of “family” in ICU care is not fixed but socially constructed through institutional practices and communication processes. Furthermore, this shift reflects a move toward a relational understanding of family, where emotional, moral, and decisional connections may take precedence over biological or spatial proximity (Holstein and Gubrium 1995; Secunda and Kruser 2022).

In Japan, the traditional description of “family” in healthcare settings has typically depended on the ability to physically visit hospitals. Consequently, HCPs have identified family members who frequently visited and maintained close relationships as “key persons,” interacting primarily with them (Miyanaga and Poudyal 2019). Surrogate decision-makers have traditionally been selected from among these actively involved family members. However, the effective implementation of remote visits has allowed patients to maintain connections with family members they personally prefer, regardless of physical visitation constraints. This advancement impacts surrogate decision-making processes by enabling a more patient-centered approach rather than relying exclusively on HCP perceptions. Consequently, HCPs are increasingly encouraged to redefine their concept of “family,” promoting a more inclusive, patient-oriented framework for family engagement and surrogate decision-making in ICU practices.

### Limitations

This study has some limitations. First, participants were recruited exclusively from a central COVID-19 treatment facility in the Kansai region of Japan. As such, the findings may not be fully generalized to ICU settings in other regions. However, because Japan has a universal health insurance system and a relatively standardized nationwide healthcare infrastructure, regional disparities in medical care are less pronounced. Therefore, the findings may still provide useful insights for other large urban hospitals in Japan but may not reflect the circumstances of smaller or rural institutions with greater limitations in staffing and resources. Second, there is the possibility of selection bias because participation was voluntary and those who were more reflective or motivated to share their experiences may have been more likely to participate. In addition, the participants’ recollections may have been affected by recall bias—that is, their memories of the early pandemic may have become less vivid or reframed in a more positive or reflective way over time (Sun et al. 2020). Therefore, the perspectives captured in this study should be understood within this contextual limitation.

## Conclusions

This study focused on the impact of the COVID-19 pandemic on communication and care among HCPs and patients and their families in Japanese ICUs. Through the interviews, nine important themes were identified. During the pandemic, HCPs faced challenges in communication due to infection-control measures and visitation restrictions that created significant communication barriers; interdisciplinary collaboration was strengthened, and nurses’ essential roles in supporting family interactions were reaffirmed. Additionally, the introduction of remote visits expanded the traditional understanding of “family,” encouraging more patient-centered surrogate decision-making. These pandemic experiences offer valuable insights for improving communication practices, supporting flexible visitation policies, and promoting patient- and family-centered approaches, both during future healthcare crises and in routine ICU care. From a practical perspective, enhancing interdisciplinary cooperation through regular interdisciplinary conferences and structured communication training, while also reinforcing the central role of nurses in facilitating family communication and emotional support, may help advance the quality of ICU practice.

## Supplementary Information

Below is the link to the electronic supplementary material.

ESM (DOCX 28.0 KB)

## Data Availability

The data are not publicly available in order to protect participant confidentiality. Public disclosure of the data would conflict with the ethical agreement signed by the participants and approved by the Osaka University Clinical Research Review Committee. However, access to certain aggregated data may be granted upon approval by the Review Committee. For inquiries, please contact the Osaka University Clinical Research Review Committee at: rinri@hp-crc.med.osaka-u.ac.jp.
