# Supplementary material for: Reflections and Practical Insights on Communication and Care for Patients and Families in Japanese Intensive Care Units During COVID-19: A Semi-structured Interview Study of Healthcare Providers
Source: Asian Bioeth Rev. 2026 Apr 25;18(3):549–67. doi: 10.1007/s41649-025-00402-z (PMC13350776; doi:10.1007/s41649-025-00402-z)
Supplement: Supplementary file 1 — (DOCX 28.0 KB) [file 41649_2025_402_MOESM1_ESM.docx]

**Supplementary Information**

**TABLES**

**Table S1. Examples of Coded Quotations**

|  | **Illustrative Quotation** | **Code** |
| --- | --- | --- |
| **Example 1** | Recognizing that individual HCPs could not sustain prolonged patient care, we standardized manuals for intubation, extubation, and management before initiating COVID-19 admissions. | Standardizing patient management for seamless handoffs |
| **Example 2** | If we could have devised a system more quickly or found innovative methods for visits, we could have created more opportunities for patients and families to connect. | Missed opportunities for patient–family connection owing to delayed innovation |
